# Development and validation of a simple risk scoring system for a COVID-19 diagnostic prediction model

**DOI:** 10.5578/tt.20239601

**Published:** 2023-12-04

**Authors:** Özge AYDIN GÜÇLÜ, Ahmet URSAVAŞ, Gökhan OCAKOĞLU, Nilüfer Aylin ACET ÖZTÜRK, Dilara ÖMER TOPÇU, Orkun Eray TERZİ, Uğur ÖNAL, Aslı GÖREK DİLEKTAŞLI, İmran SAĞLIK, Funda COŞKUN, Dane EDİGER, Esra UZASLAN, Halis AKALIN, Mehmet KARADAĞ

**Affiliations:** 1 Department of Pulmonary Diseases, Uludağ University Faculty of Medicine, Bursa, Türkiye; 2 Department of Biostatistics, Uludağ University Faculty of Medicine, Bursa, Türkiye; 3 Department of Infectious Diseases and Clinical Microbiology, Uludağ University Faculty of Medicine, Bursa, Türkiye

## Abstract

**ABSTRACT**

**
Development and validation of a simple risk scoring system for a COVID-19 diagnostic prediction model
**

**Introduction:** *In a resource-constrained situation, a clinical risk stratification system can assist in identifying individuals who are at higher risk and should be tested for COVID-19. This study aims to find a predictive scoring model to estimate the COVID-19 diagnosis.
*

**Materials and Methods:** *Patients who applied to the emergency pandemic clinic between April 2020 and March 2021 were enrolled in this retrospective study. At admission, demographic characteristics, symptoms, comorbid dis- eases, chest computed tomography (CT), and laboratory findings were all recorded. Development and validation datasets were created. The scoring system was performed using the coefficients of the odds ratios obtained from the multivariable logistic regression analysis.
*

**Results:** *Among 1187 patients admitted to the hospital, the median age was 58 years old (22-96), and 52.7% were male. In a multivariable analysis, typi- cal radiological findings (OR= 8.47, CI= 5.48-13.10, p< 0.001) and dyspnea (OR= 2.85, CI= 1.71-4.74, p< 0.001) were found to be the two important risk factors for COVID-19 diagnosis, followed by myalgia (OR= 1.80, CI= 1.08- 2.99, p= 0.023), cough (OR= 1.65, CI= 1.16-2.26, p= 0.006) and fatigue
*

*
symptoms (OR= 1.57, CI= 1.06-2.30, p= 0.023). In our scoring system, dysp- nea was scored as 2 points, cough as 1 point, fatigue as 1 point, myalgia as 1 point, and typical radiological findings were scored as 5 points. This scoring system had a sensitivity of 71% and a specificity of 76.3% for a cut-off value of >2, with a total score of 10 (p< 0.001).
*

**Conclusion:** *The predictive scoring system could accurately predict the diag- nosis of COVID-19 infection, which gave clinicians a theoretical basis for devising immediate treatment options. An evaluation of the predictive efficacy of the scoring system necessitates a multi-center investigation.
*

**Key words:** *COVID-19; scoring system; prediction model; diagnosis
*

**ÖZ**

**
COVID-19 tanısal tahmin modeli için basitleştirilmiş risk skorlama sisteminin geliştirilmesi ve doğrulanması
**

**Giriş:** *Kaynakların kısıtlı olduğu bir durumda klinik risk skorlama sistemi, daha yüksek risk altında olan ve COVID-19 için test edilme- si gereken bireylerin belirlenmesine yardımcı olabilir. Bu çalışmanın amacı, COVID-19 tanısını tahmin edebilecek öngörücü bir skor- lama modeli bulmaktır.
*

**Materyal ve Metod:** *Çalışmaya Nisan 2020 ile Mart 2021 tarihleri arasında acil pandemi polikliniğine başvuran hastalar dahil edilmiş- tir. Başvuru sırasında olguların demografik özellikleri, semptomları, komorbid hastalıkları, toraks bilgisayarlı tomografi (BT) ve labora- tuvar bulguları retrospektif olarak değerlendirilmiştir. Geliştirme ve doğrulama veri setleri oluşturulmuştur. Çok değişkenli lojistik reg- resyon analizi sonucunda elde edilen katsayılar kullanılarak skorlama sistemi gerçekleştirilmiştir.
*

**Bulgular:** *Hastaneye başvuran 1187 hastanın ortanca yaşı 58’di (22-96) ve %52,7’si erkekti. Çok değişkenli analizde, tipik radyolojik bulgular (OR= 8,47, CI= 5,48-13,10, p< 0.001) ve dispne (OR= 2,85, CI= 1,71-4,74, p< 0,001) COVID-19 tanısı için iki önemli risk faktörü olarak bulunmuş, bunları miyalji (OR= 1,80, CI= 1,08-2,99, p= 0,023), öksürük (OR= 1,65, CI= 1,16-2,26, p= 0,006) ve yorgunluk semptomları (OR= 1,57, CI= 1,06-2,30, p= 0,023) izlemiştir. Skorlama sistemimizde dispne 2 puan, öksürük 1 puan, yor- gunluk 1 puan, miyalji 1 puan ve tipik radyolojik bulgular 5 puan olarak değerlendirilmiştir. Toplam skor 10 ve >2 cut off değeri için bu skorlama sisteminin duyarlılığı %71, özgüllüğü ise %76,3 olarak bulunmuştur (p< 0,001).
*

**Sonuç:** *Tanısal öngörücü skorlama sistemi COVID-19 enfeksiyonu tanısını doğru bir şekilde tahmin edebilmiş ve bu da klinisyenlere acil tedavi seçenekleri sunmaları için teorik bir temel sağlamıştır. Skorlama sisteminin öngörücü etkinliğinin değerlendirilmesi için çok merkezli bir araştırmaya ihtiyaç vardır.
*

**Anahtar kelimeler:** *COVID-19; skorlama sistemi; tahmin modeli; tanı
*

## INTRODUCTION

A new Coronavirus (CoV) with clinical features comparable to SARS CoV-1 (SARS-CoV-1) and Middle East Respiratory Syndrome (MERS) CoV (MERS-CoV) emerged at the end of 2019 (1). This new CoV type, SARS-CoV-2, rapidly spread worldwide, with the first case identified on March 11, 2020, in Türkiye. As of September 21, 2022, there were 161.852.382 verified COVID-19 cases and 101.068 deaths.

Polymerase chain reaction (PCR) testing is, therefore, the gold standard for identifying and establishing a patient’s COVID-19 viral infection (2). However, this type of diagnostic examination has several drawbacks and limits. It has been demonstrated, for instance, that upper respiratory tract samples contain the maximum viral loads three days following the onset of symptoms and that the results of PCR testing take at least one day to be obtained after sampling (3). As the SARS-CoV-2 pandemic spreads worldwide, we require improved diagnostic screening technologies that are rapid, accurate, validated, and broadly accessible.

The pandemic of COVID-19 has had a severe negative impact on Türkiye and the rest of the world. The capacity of hospitals in Türkiye to triage, identify, and treat COVID-19 patients has decreased since the onset of the COVID-19 pandemic. Improving hospital screening and classifying individuals at high risk of infection is critical for rapid and appropriate isolation, treatment, and use of limited health resources. There

is no validated, widely available risk stratification system to assist clinicians in deciding when COVID-

19 diagnostic testing is required. A clinical risk stratification approach can assist in identifying high- risk individuals who should be tested for COVID-19 when resources are limited. This study aims to identify clinical, radiographic, and laboratory parameters capable of predicting the presence or absence of COVID-19 infection. The goal is to develop and validate a diagnostic model that effectively selects individuals at risk for COVID-19 in a suitable and safe manner.

## MATERIALS and METHODS

Patients who applied to the emergency pandemic clinic between April 2020 and March 2021 were enrolled in this retrospective study. At admission, demographic characteristics, symptoms, comorbid diseases, chest computed tomography (CT), and laboratory findings were all recorded. Before the COVID-19 patient’s admission, comorbidities were those that had been diagnosed. Baseline ferritin, C-reactive protein (CRP), D-dimer, lymphocyte, and eosinophil values were obtained.

Symptomatic cases aged 18 years and older who applied to the emergency pandemic clinic were included in the study. Patients who were transported immediately to the critical care unit and those who did not undergo thorax computed tomography were excluded from the study. Figure 1 provides a summary of the study protocol.

Aydın Güçlü Ö, Ursavaş A, Ocakoğlu G, Demirdöğen E, Acet Öztürk NA, Ömer Topçu D, et al.

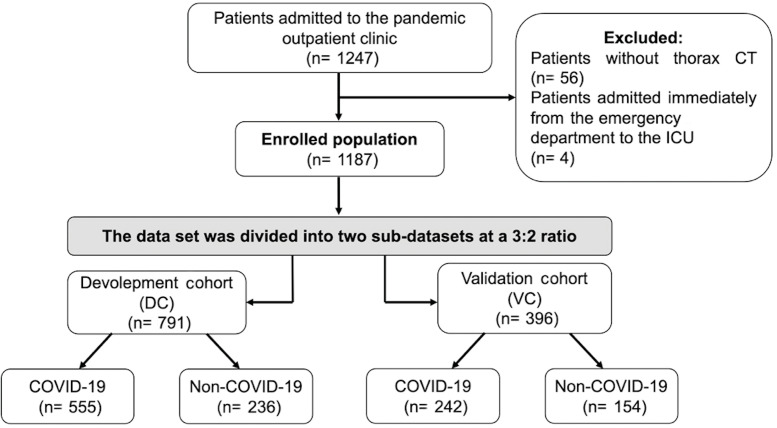
** Figure 1.** Study flow chart.

The study was authorized by the Uludağ University Faculty of Medicine Clinical Research Ethics Committee (Approval No: 2020-19/7), the Ministry of Health’s Ethical Committee, and adhered to the principles of the Helsinki Declaration.

## Definitions

Possible COVID-19 cases have been identified by national guidelines issued by the Republic of Türkiye Ministry of Health. When patients were admitted, nasopharyngeal swabs were taken for real-time reverse transcriptase-polymerase chain reaction testing (RT-PCR).

According to the expert consensus statement of the Radiological Society of North America (RSNA), chest CT patterns are classified as “negative for pneumonia,” “indeterminate appearance,” “atypical appearance,” and “typical appearance.” (4). In our study, an expert pulmonologist and a chest radiologist examined the chest CT of each suspected COVID-19 patient. The

Roche Elecsys® Anti-SARS-CoV-2 immunological test

was utilized to detect IgG antibodies against SARS- CoV-2 in serum samples from cases with negative SARS-COV-2-PCR and clinical and radiological suspicion of COVID-19 disease. The test, reported to calculate 95% sensitivity, 100% specificity, and positive and negative predictive values approaching

100% in diagnosing SARS-COV-2, was studied from serum samples taken at least two weeks after the disease of unvaccinated patients (5). Cases were categorized as “definite COVID-19 positive” if they tested positive for SARS-COV-2-PCR or if their PCR test was negative but the antibody test yielded a positive result.

## Statistical Analysis

The development group (n= 791) and the validation group (n= 396) were separated into two sub-datasets at a 3:2 ratio from the entire data set (n= 1187). Clinical features were compared between COVID-19 and non-COVID-19 patient groups within each development and validation group. The Shapiro-Wilk test was used to determine if continuous variables conformed to the normal distribution. Since continuous variables did not follow a normal distribution, they were presented with the median (minimum: maximum), whereas categorical variables were provided with frequency and the accompanying percentage values. The Mann-Whitney U test was used to compare continuous data between groups, while the Chi-square and Fisher’s exact tests were used to compare categorical variables. A univariate logistic regression analysis (LRA) was done on a development cohort to find factors that could affect the state of COVID-19. The multivariable logistic

regression analysis was performed using variables that met the p< 0.25 threshold as determined by the univariate logistic regression analysis. The coefficients derived from the logistic regression model were utilized to formulate risk score models. In the validation group, three risk score models were developed and validated.

Three risk scores were developed based on the coefficients of the final model. The scoring system used the coefficients of the odds ratios obtained from the multivariable logistic regression analysis (Model 1). The relevant coefficients have been rounded to the nearest integer (Model 2 and Model 3). The area under the receiver operating characteristic curve (AUC) was calculated for each of the risk score models. SPPS (IBM Corp. 2012 release). IBM SPSS Statistics for Windows, Version 21.0, Armonk, New York: IBM Corp. was utilized to conduct the statistical analysis. The type I error rate for statistical analysis was set at 5%.

## RESULTS

During the research period, a total of 1.247 individuals were admitted to the pandemic emergency clinic. The study excluded 56 patients who did not have a chest CT scan and four patients who were directly referred to the critical care unit. Of the 1187 hospitalized patients, 797 (67.1%) tested positive for SARS-COV-2-PCR, while 390 (32.9%) did not.

Twenty-one patients with negative PCR results had positive antibody tests. Table 1 describes the patients’ characteristics. In the overall population, the median age was 58 (22-96), and 52.7% were male. There was at least one comorbid disease in 563 (47.4%) cases. Hypertension (28.9%), diabetes mellitus (17.7%), and coronary artery disease (11.1%) were the most prevalent comorbid diseases. The dataset was divided into separate development and validation datasets. Out of the 791 patients assigned to the development cohort, 555 individuals (70.5%) were identified as COVID-19-positive. Three hundred ninety-six patients were appointed to the validation cohort, of which 242 (61.1%) tested positive.

The most common symptoms in the patients were cough (49.8%), fatigue (33.4%) and dyspnea (24.1%), respectively. Cough, fatigue, and dyspnea were more common symptoms in COVID-19 patients as compared to non-COVID-19 cases in both the

development cohort (p< 0.001, p= 0.03, p= 0.006, respectively) and the validation cohort (p< 0.001, p= 0.001, p= 0.011, respectively). Radiological findings were “typical” in 49% of the cases and “negative for pneumonia” in 30.7%. Table 1 shows each group’s clinical and demographic information at baseline.

In both the development and validation cohorts, it was shown that typical COVID-19 radiological findings were statistically significant in COVID-19 patients compared to non-COVID-19 cases (both, p< 0.001). When compared to non-COVID-19 cases in both the development (p= 0.003, p< 0.001, p< 0.001, p< 0.001, respectively) and validation cohorts (p= 0.025, p= 0.001, p< 0.001, p< 0.001,

respectively), higher CRP and ferritin levels, as well as lower lymphocyte and eosinophil levels, were found to be statistically significant in COVID-19 patients (Table 1).

The association between dyspnea and potential confounding comorbid diseases that may affect dyspnea symptoms was examined in univariate analyses. It has been shown that there is no statistically significant association between dyspnea symptoms with congestive heart failure [6 (3.4%) vs 169

(96.6%)], asthma [15 (8.6%) vs 160 (91.4%)], COPD

[10 (5.4%) vs 165 (94.3%)], or chronic kidney failure

[6 (3.4%) vs 169 (96.6%)]) (p= 0.114, p= 0.620,

p= 0.267, p= 0.248, respectively). In a multivariable analysis, typical radiological findings (OR= 8.47, CI= 5.48-13.10, p< 0.001) and dyspnea (OR= 2.85,

CI= 1.71-4.74, p< 0.001) were found to be the two important risk factors for COVID-19 diagnosis, followed by myalgia (OR= 1.80, CI= 1.08-2.99, p= 0.023), cough (OR= 1.65, CI= 1.16-2.26,

p= 0.006) and fatigue symptoms (OR= 1.57, CI= 1.06-2.30, p= 0.023) (Table 2).

In the initial model, dyspnea was allocated 3 points, cough 2 points, fatigue 2 points, myalgia 2 points, and typical radiological findings in Thorax CT were assigned 8 points. The risk score resulting from the relevant scoring achieved 0.79 AUC in the development cohort, 0.81 AUC in the validation cohort, and 0.80 AUC in the overall patients. Conversely, the proposed second and third scoring systems involve adjusting the coefficients of the variables in the model based on total scores, setting them to 10 and 19, respectively (Models 2 and 3).

**Table:** 

Table 1. The baseline characteristics of hospitalized patients in the development and validation cohorts							
		Dev	elopment cohort		Validation cohort		
							
			(n= 791)		(n= 396)		
							
Overall population (n= 1187)		Non-COVID	COVID		Non-COVID	COVID	
							
		(n= 236)	(n= 555)	p	(n= 154)	(n= 242)	p
Age, years	58 (18-96)	56 (22-96)	58 (18-93)	0.680†	59 (18-88)	58.50 (21-93)	0.753†
Gender, male	625 (52.70%)	119 (50.40%)	261 (47%)	0.382‡	78 (50.60%)	104 (43%)	0.135‡
Comorbidity, n (%)	563 (47.40%)	111 (47%)	260 (46.80%)	0.962‡	74 (48.10%)	118 (48.80%)	0.891‡
Hypertension	343 (28.90%)	57 (24.20%)	169 (30.50%)	0.073‡	36 (23.40%)	81 (33.50%)	0.032‡
Diabetes mellitus	210 (17.70%)	47 (19.90%)	95 (17.10%)	0.348‡	28 (18.20%)	40 (16.50%)	0.671‡
Coronary artery disease	140 (11.80%)	22 (9.30%)	66 (11.90%)	0.293‡	18 (11.70%)	34 (14%)	0.498‡
COPD	51 (4.30%)	10 (4.20%)	21 (3.80%)	0.764‡	6 (3.90%)	14 (5.80%)	0.403‡
Asthma	82 (6.90%)	11 (4.70%)	44 (7.90%)	0.098‡	8 (5.20%)	19 (7.90%)	0.307‡
Malignancy	79 (6.70%)	21 (8.90%)	26 (4.70%)	0.022‡	22 (14.30%)	10 (4.10%)	<0.001‡
Chronic renal failure	27 (1.20%)	7 (3%)	8 (1.40%)	0.161§	4 (2.60%)	8 (3.30%)	0.772§
Chronic liver failure	24 (2%)	1 (0.40%)	3 (0.50%)	>0.99§	0	1 (0.40%)	>0.99§
Baseline vital signs, n (%)							
Fever	341 (28.70%)	62 (26.30%)	148 (26.70%)	0.908‡	45 (29.20%)	86 (35.50%)	0.193‡
Throat ache	120 (10.10%)	29 (12.30%)	54 (9.70%)	0.283‡	14 (9.10%)	23 (9.50%)	0.890‡
Dyspnea	286 (24.10%)	25 (10.60%)	156 (28.10%)	<0.001‡	15 (9.70%)	90 (37.20%)	<0.001‡
Cough	591 (49.80%)	109 (46.20%)	303 (54.60%)	0.030‡	53 (34.40%)	126 (52.10%)	0.001‡
Fatigue	397 (33.40%)	63 (26.70%)	204 (36.80%)	0.006‡	39 (25.30%)	91 (37.60%)	0.011‡
Diarrhea	65 (5.50%)	15 (6.40%)	30 (5.40%)	0.597‡	10 (6.50%)	10 (4.10%)	0.296‡
Myalgia	195 (16.40%)	28 (11.90%)	96 (17.30%)	0.054‡	29 (18.80%)	42 (17.40%)	0.709‡
Smell and taste dysfunction	61 (5.10%)	23 (9.70%)	21 (3.80%)	0.001‡	11 (7.10%)	6 (2.50%)	0.026‡
Chest CT images, n (%)							
Typical	582 (49%)	43 (18.20%)	361 (65%)	<0.001‡	25 (16.20%)	153 (63.20%)	<0.001‡
Indeterminate	154 (13%)	39 (16.50%)	51 (9.20%)		28 (18.20%)	36 (14.90%)	
Atypical	87 (7.30%)	29 (12.30%)	29 (5.20%)		22 (14.30%)	7 (2.90%)	
Negative	364 (30.70%)	125 (53%)	114 (20.50%)		79 (51.30%)	46 (19%)	
Initial laboratory findings							
C-reactive protein, mg/L	22.10 (0.20-15035)	11 (0.20-414)	27.15 (0.20-2916)	0.003†	15.55 (0.20-15035)	27 (0.20-35.20)	0.025†
D-dimer, mg/L	0.57 (0-967)	0.63 (0-28)	0.56 (0.17-967)	0.218†	0.59 (0.10-44.85)	0.54 (0.10-49)	0.511†
Ferritin, ng/mL	147.60 (2-11080)	95 (2-2843)	187.70 (4.90-11080)	<0.001†	107.15 (2-2431)	170.70 (2.67-4351)	0.001†
Lymphocyte, per mm3	1510 (80-16402.50)	1750 (95.30-5295)	1390 (80-16402.50)	<0.001†	1775.50 (113-10470)	1387 (120-12250)	<0.001†
Eosinophil, per mm3	10 (0-4025)	41 (0-4025)	5 (0-1200)	<0.001†	39.50 (0-910)	4 (0-1010)	<0.001†
Data were presented as median (minimum-maximum) and n (%).							
							
†: Mann-Whitney U test, ‡: Chi-square test, §: Fisher’s exact test.							

**Table:** 

Table 2. The outcomes of univariable and multivariate logistic regression analyses							
		Univariable analysis			Multivariable analysis		
	Crude OR	95% CI	p	Adjusted OR	95% CI	p	
Age, years	0.99	0.98-1.01	0.474	-	-	-	
Gender, male	0.87	0.64-1.18	0.382	-	-	-	
Symptoms							
Fever	1.02	0.72-1.44	0.908	-	-	-	
Throat ache	0.77	0.48-1.24	0.284	-	-	-	
Dyspnea	3.30	2.10-5.20	<0.001	2.85	1.71-4.74	<0.001	
Cough	1.04	1.03-1.90	0.031	1.65	1.16-2.36	0.006	
Fatigue	1.60	1.14-2.23	0.006	1.57	1.06-2.30	0.023	
Myalgia	1.55	0.99-2.44	0.056	1.80	1.08-2.99	0.023	
Chest CT images, n (%)			<0.001			<0.001	
Typical	9.21	6.14-13.81	<0.001	8.47	5.48-13.10	<0.001	
Indeterminate	1.43	0.88-2.34	0.148	1.53	0.91-2.59	0.111	
Atypical	1.10	0.62-1.95	0.753	1.10	0.60-2.01	0.768	
Initial laboratory findings							
Lymphocyte, per mm3	1	0.99-1.01	0.722				
C-reactive protein, mg/L	1.01	1-1.01	0.083	0.99	0.98-1.01	0.148	
D-dimer, mg/L	1.01	0.99-1.02	0.200	1.01	0.99-1.02	0.844	
Ferritin, ng/mL	1	1-1.01	0.009	-	-	-	
Significance for the multivariable model is p< 0.001, and significance for Hosmer and Lemeshow test is p= 0.494.

The second model had an AUC of 0.79 in the development cohort, 0.81 in the validation cohort, and 0.80 in the overall population, whereas the third scoring system had an AUC of 0.79 in the development cohort, 0.81 in the validation cohort, and 0.80 in the overall population (Table 3). Model 2 was identified as the final model because it had similar sensitivity and specificity as the other models and was applicable, practical, and easy to remember. Table 4 shows the sensitivity, specificity, and positive and negative predictive values for various cut-off values for Model 2.

## DISCUSSION

This study aimed to evaluate clinical, radiographic, and laboratory factors that can predict the presence or absence of COVID-19 infection to develop and validate a diagnostic model for identifying people at risk for COVID-19.

In the initial scoring model we developed, dyspnea was allocated 3 points, cough 2 points, fatigue 2 points, myalgia 2 points, and typical radiological findings in Thorax CT were assigned 8 points. When

the corresponding scoring was evaluated out of 17, it yielded a risk score of 0.79 AUC for the development cohort, 0.81 AUC for the validation cohort, and 0.80 AUC for the overall population. The coefficients of the model variables were adjusted to set them as 10 and 19, respectively, over the total scores in the second and third scoring systems. Model 2 was identified as the final model because it had similar sensitivity and specificity as the other models and was applicable, practical, and easy to remember.

In both the development and validation cohorts, cough, fatigue, and dyspnea were more prevalent in COVID-19 patients than in non-COVID-19 cases. The main symptoms of COVID-19, according to the Centers for Disease Control and Prevention, are high temperature, coughing, dyspnea, fatigue, musculoskeletal pain, headaches, loss of smell or taste, throat pain, vomiting or nausea, and diarrhea (6).

Compared to non-COVID-19 cases, it was determined that typical COVID-19 radiological findings in COVID-19 patients were statistically significant in both the development and validation cohorts.

**Table:** 

Table 3. Receiver operating characteristic analysis of COVID-19 risk scores							
	Model 1	Model 2	Model 3				
Dyspnea	3 points	2 points	4 points				
Cough	2 points	1 point	2 points				
Fatigue	2 points	1 point	2 points				
Myalgia	2 points	1 point	2 points				
Typical chest CT images	8 points	5 points	9 points				
	17 points	10 points	19 points				
Development cohort							
AUC (95% CI)	0.79 (0.76-0.82)	0.79 (0.76-0.82)	0.79 (0.76-0.82)				
Cut-off point	>4	>2	>4				
Sensitivity	71%	71%	71%				
Specificity	76.30%	76.30%	76.30%				
p	<0.001	<0.001	<0.001				
Validation cohort							
AUC (95% CI)	0.81 (0.77-0.85)	0.81 (0.77-0.85)	0.81 (0.77-0.85)				
Cut-off point	>3	>2	>4				
Sensitivity	78.50%	69%	69%				
Specificity	72.10%	81.20%	81.20%				
p	<0.001	<0.001	<0.001				
Total							
AUC (95% CI)	0.80 (0.77-0.82)	0.80 (0.77-0.82)	0.80 (0.77-0.82)				
Cut-off point	>4	>2	>4				
Sensitivity	70.40%	70.39%	70.39%				
Specificity	78.20%	78.21%	78.21%				
p	<0.001	<0.001	<0.001				

**Table:** 

Table 4. Sensitivity, specificity, and predictive values for Model 2 cut-off values								
	"Development cohort	Validation cohort"							
								
Risk score	Sensitivity (%)	Specificity (%)	PPV (%)	NPV (%)	Sensitivity (%)	Specificity (%)	PPV (%)	NPV (%)
>0	95.14	27.54	75.50	70.70	95.45	25.97	67	78.40
>1	81.62	63.98	84.20	59.70	80.58	64.94	78.30	68
>2	70.99	76.27	87.60	52.80	69.01	81.17	85.20	62.50
>3	66.85	78.39	87.90	50.10	64.46	83.77	86.20	60
>4	65.59	81.36	89.20	50.10	63.64	83.77	86	59.40
>5	60.72	85.17	90.60	48	57.85	87.01	87.50	56.80
>6	32.79	93.64	92.40	37.20	38.84	94.81	92.20	49.70
>7	12.43	97.03	90.80	32	20.66	99.35	98	44.30
>8	3.60	99.58	95.20	30.50	6.20	100	100	40.40
>9	0.36	100	100	29.90	0.83	100	100	39.10
PPV: Positive predictive value, NPV: Negative predictive value.

COVID-19 imaging characteristics have been observed to have a high sensitivity, particularly in high-prevalence areas (7). Hu et al. found that 50% of asymptomatic SARS-CoV-2 cases had typical ground-glass opacities, and 20% had atypical CT appearance (8). When RT-PCR was used as the gold standard, thorax CT had 97% sensitivity, 25% specificity, and 68% accuracy in detecting COVID- 19 infection (9).

In both the development and validation cohorts, greater CRP and ferritin levels and reduced lymphocyte and eosinophil levels were found to be statistically significant in COVID-19 patients compared to non-COVID-19 cases. Lymphopenia and an increase in CRP, ferritin, and D-dimer are typical laboratory abnormalities observed; some of which indicate disease severity (10,11). Lymphopenia and eosinopenia are associated with increased disease severity and a poor prognosis (12). Several factors contribute to lymphopenia, including the cytotoxic effects of the virus, the induction of apoptosis, IL1-mediated pyroptosis, and the inhibition of bone marrow by inflammatory cytokines (13). Several reports have suggested lymphopenia as a strong indicator of COVID-19 infection (14-16).

The multivariable analysis revealed that, in diagnosing COVID-19, the presence of typical radiological features increased the risk by eight times, dyspnea by three times, myalgia by two times, cough by two times, and fatigue by 1.5 times. Clinical examinations and radiological diagnostics proved to be valuable diagnostic approaches, especially during the initial phases of the pandemic when confirmed molecular and serological testing options were not available (17,18). Kovács et al. demonstrated the sensitivity, specificity, and accuracy of RT-PCR, using thorax CT as the gold standard, as 65%, 83%, and 67%, respectively, as per the inverse calculation approach (19).

Testing techniques widely used for diagnosing COVID-19 include viral nucleic acid testing, computed tomography scans, and antigen testing (20,21). In the initial week of a suspected infection, both serological and molecular tests become ineffective due to the virus being in its incubation phase and resulting in insufficient copies of viral RNA present in circulation (22,23). The time lapse between sample collection and result retrieval often exceeds 24 hours, and it is recognized that testing

samples from the upper respiratory tract can yield a false-negative rate (24). Effective acute care, infection control, and avoidance of nosocomial transmission all depend on a rapid COVID-19 diagnosis upon admission. The burden that periodic increases in the incidence of COVID-19 have placed on health systems worldwide highlights the importance of accurate early risk classification in the general population. In the absence of laboratory testing for SARS-CoV-2, Our diagnostic prediction model was designed for use by healthcare professionals to facilitate the clinical diagnosis of patients with COVID-19 and to support infection treatment decisions within the initial 24 hours of admission.

## Limitations

Our study exhibits certain limitations. It is an observational study relying on data obtained from health records due to the impracticality of conducting in-person visits and interviews amid the pandemic. This research was retrospective, wherein symptom reporting was voluntary. This may have concluded as the response bias. The patients were asked about symptoms in a way that allowed them to indicate whether or not they were present subjectively. No specific symptom scales were employed. In our study, the nonspecific and subjective fatigue symptom was questioned in the pandemic outpatient clinic without a scale, and possible contributing factors, including anemia, undiagnosed sleep apnea syndrome, comorbidities, and medication therapy, were not assessed. Finally, the predictive performance of the models can also be influenced by the phase of the disease. Although we aimed to mitigate this effect by exclusively analyzing patients in the emergency department, the time lapse between the onset of symptoms could also be a contributing factor.

## CONCLUSION

The predictive scoring system accurately predicted the diagnosis of COVID-19 infection, which gave clinicians a theoretical basis for devising immediate treatment options. However, to fully evaluate the predictive effectiveness of the scoring system, it must be externally validated in a multi-center study.

**Ethical Committee Approval:** This study was approved by the Uludağ University Faculty of Medicine Clinical Research Ethics Committee (Decision no: 2020-19/7, Date: 04.11.2020).

## CONFLICT of INTEREST

The authors declare that they have no conflict of interest.

## AUTHORSHIP CONTRIBUTIONS

Concept/Design: ÖAG, AU, GO, ED Analysis/Interpretation: GO, ÖAG

Data acqusition: NAAÖ, DÖT, OET, UÖ, AGD, İS, FC, DE, EU, MK

Writing: ÖAG, AU

Clinical Revision: ÖAG, AU, HA

Final Approval: DE, ÖAG, AU, GO, ED, AGD, FC

## REFERENCES

1. *
  Velavan TP, Meyer CG. The COVID‐19 epidemic. Trop Med Int Health 2020; 25(3): 278. https://doi.org/10.1111/ tmi.13383*
2. *
  Tahamtan A, Ardebili A. Real-time RT-PCR in COVID-19 detection: Issues affecting the results. Expert Rev Mol Diagn 2020; 20(5): 453-4. https://doi.org/10.1080/1473 7159.2020.1757437*
3. *
  To KKW, Tsang OTY, Leung WS, Tam AR, Wu TC, Lung DC, et al. Temporal profiles of viral load in posterior oropha- ryngeal saliva samples and serum antibody responses during infection by SARS-CoV-2: An observational cohort study. Lancet Infect Dis 2020; 20(5): 565-74. https://doi. org/10.1016/S1473-3099(20)30196-1*
4. *
  O’Neill SB, Byrne D, Müller NL, Jalal S, Parker W, Nicolaou S, et al. Radiological Society of North America (RSNA) expert consensus statement related to chest CT findings in COVID-19 versus CO-RADS: Comparison of reporting system performance among chest radiologists and end-us- er preference. Can Assoc Radiol J 2021; 72(4): 806-13. https://doi.org/10.1177/0846537120968919*
5. *
  Chan CW, Parker K, Tesic V, Baldwin A, Tang NY, van Wijk XMR, et al. Analytical and clinical evaluation of the auto- mated elecsys anti-SARS-CoV-2 antibody assay on the roche cobas e602 analyzer. Am J Clin Pathol 2020; 154(5): 620-6. https://doi.org/10.1093/ajcp/aqaa155*
6. *
  Wu Z, McGoogan JM. Characteristics of and important lessons from the Coronavirus disease 2019 (COVID-19) outbreak in China: Summary of a report of 72 314 cases from the Chinese Center for Disease Control and Prevention. JAMA 2020; 323(13): 1239-42. https://doi. org/10.1001/jama.2020.2648*
7. *
  Xu B, Xing Y, Peng J, Zheng Z, Tang W, Sun Y, et al. Chest CT for detecting COVID-19: A systematic review and meta-analysis of diagnostic accuracy. Eur Radiol 2020; 30(10): 5720-7. https://doi.org/10.1007/s00330-020- 06934-2*
8. *
  Hu Z, Song C, Xu C, Jin G, Chen Y, Xu X, et al. Clinical characteristics of 24 asymptomatic infections with COVID- 19 screened among close contacts in Nanjing, China. Sci China Life Sci 2020; 63(5): 706-11. https://doi. org/10.1007/s11427-020-1661-4*
9. *
  Ai T, Yang Z, Hou H, Zhan C, Chen C, Lv W, et al. Correlation of chest CT and RT-PCR testing for Coronavirus disease 2019 (COVID-19) in China: A report of 1014 cases. Radiol 2020; 296(2): E32-E40. https://doi. org/10.1148/radiol.2020200642*
10. *
  Terpos E, Ntanasis‐Stathopoulos I, Elalamy I, Kastritis E, Sergentanis TN, Politou M, et al. Hematological findings and complications of COVID‐19. Am J Hematol 2020; 95(7): 834-47. https://doi.org/10.1002/ajh.25829*
11. *
  Zhou F, Yu T, Du R, Fan G, Liu Y, Liu Z, et al. Clinical course and risk factors for mortality of adult inpatients with COVID-19 in Wuhan, China: A retrospective cohort study. Lancet 2020; 395(10229): 1054-62. https://doi. org/10.1016/S0140-6736(20)30566-3*
12. *
  Zhao L, Zhang YP, Yang X, Liu X. Eosinopenia is associated with greater severity in patients with Coronavirus disease 2019. Allergy 2021; 76(2): 562. https://doi.org/10.1111/ all.14455*
13. *
  Huang I, Pranata R, Lim MA, Oehadian A, Alisjahbana B. C-reactive protein, procalcitonin, D-dimer, and ferritin in severe Coronavirus disease-2019: A meta-analysis. Ther Adv Respir Dis 2020; 14: 1753466620937175. https:// doi.org/10.1177/1753466620937175*
14. *
  Azkur AK, Akdis M, Azkur D, Sokolowska M, van de Veen W, Brüggen MC, et al. Immune response to SARS‐CoV‐2 and mechanisms of immunopathological changes in COVID‐19. Allergy 2020; 75(7): 1564-81. https://doi. org/10.1111/all.14364*
15. *
  Kermali M, Khalsa RK, Pillai K, Ismail Z, Harky A. The role of biomarkers in diagnosis of COVID-19-A systematic review. Life Sci 2020; 254: 117788. https://doi. org/10.1016/j.lfs.2020.117788*
16. *
  Fink D, Khan P, Goldman N, Cai J, Hone L, Mooney C, et al. Development and internal validation of a diagnostic prediction model for COVID-19 at time of admission to hospital. QJM 2021; 114(10): 699-705. https://doi. org/10.1093/qjmed/hcaa305*
17. *
  Brun AL, Gence-Breney A, Trichereau J, Ballester MC, Vasse M, Chabi ML, et al. COVID-19 pneumonia: High diagnostic accuracy of chest CT in patients with interme- diate clinical probability. Eur Radiol 2021; 31(4): 1969- 77. https://doi.org/10.1007/s00330-020-07346-y*
18. *
  Amano Y, Kage H, Tanaka G, Gonoi W, Nakai Y, Kurokawa R, et al. Diagnostic prediction of COVID-19 based on clinical and radiological findings in a relatively low COVID-19 prevalence area. Respir Investig 2021; 59(4): 446-53. https://doi.org/10.1016/j.resinv.2021.03.002*
19. *
  Kovács A, Palásti P, Veréb D, Bozsik B, Palkó A, Kincses ZT. The sensitivity and specificity of chest CT in the diagnosis of COVID-19. Eur Radiol 2021; 31(5): 2819-24. https:// doi.org/10.1007/s00330-020-07347-x*
20. *
  Pradhan M, Shah K, Alexander A, Ajazuddin, Minz S, Singh MR, et al. COVID-19: Clinical presentation and detection methods. J Immunoassay Immunochem 2022; 43(1): 1951291. https://doi.org/10.1080/15321819.2021.1951 291*
21. *
  Udugama B, Kadhiresan P, Kozlowski HN, Malekjahani A, Osborne M, Li VY, et al. Diagnosing COVID-19: The dis- ease and tools for detection. ACS Nano 2020; 14(4): 3822-35. https://doi.org/10.1021/acsnano.0c02624*
22. *
  Jarvis KF, Kelley JB. Temporal dynamics of viral load and false negative rate influence the levels of testing necessary to combat COVID-19 spread. Sci Rep 2021; 11(1): 1-12. https://doi.org/10.1038/s41598-021-88498-9*
23. *
  Rode OĐ, Kurolt IC, Puljiz I, Čivljak R, Balent NC, Laškaj R, et al. Antibody response and the clinical presentation of patients with COVID-19 in Croatia: The importance of a two-step testing approach. Eur J Clin Microbiol Infect Dis 2021; 40(2): 261-8. https://doi.org/10.1007/s10096- 020-04019-y*
24. *
  Mawaddah A, Genden HS, Lum SG, Marina MB. Upper respiratory tract sampling in COVID-19. Malays J Pathol 2020; 42(1): 23-35.
  *

